# Systems Modeling of Anti-apoptotic Pathways in Prostate Cancer: Psychological Stress Triggers a Synergism Pattern Switch in Drug Combination Therapy

**DOI:** 10.1371/journal.pcbi.1003358

**Published:** 2013-12-05

**Authors:** Xiaoqiang Sun, Jiguang Bao, Kyle C. Nelson, King Chuen Li, George Kulik, Xiaobo Zhou

**Affiliations:** 1 Department of Radiology, Wake Forest School of Medicine, Winston-Salem, North Carolina, United States of America; 2 School of Mathematical Science, Beijing Normal University, Beijing, P. R. China; 3 Department of Cancer Biology, Wake Forest School of Medicine, Winston-Salem, North Carolina, United States of America; Yong Loo Lin School of Medicine, National University of Singapore, Singapore

## Abstract

Prostate cancer patients often have increased levels of psychological stress or anxiety, but the molecular mechanisms underlying the interaction between psychological stress and prostate cancer as well as therapy resistance have been rarely studied and remain poorly understood. Recent reports show that stress inhibits apoptosis in prostate cancer cells via epinephrine/beta2 adrenergic receptor/PKA/BAD pathway. In this study, we used experimental data on the signaling pathways that control BAD phosphorylation to build a dynamic network model of apoptosis regulation in prostate cancer cells. We then compared the predictive power of two different models with or without the role of Mcl-1, which justified the role of Mcl-1 stabilization in anti-apoptotic effects of emotional stress. Based on the selected model, we examined and quantitatively evaluated the induction of apoptosis by drug combination therapies. We predicted that the combination of PI3K inhibitor LY294002 and inhibition of BAD phosphorylation at S112 would produce the best synergistic effect among 8 interventions examined. Experimental validation confirmed the effectiveness of our predictive model. Moreover, we found that epinephrine signaling changes the synergism pattern and decreases efficacy of combination therapy. The molecular mechanisms responsible for therapeutic resistance and the switch in synergism were explored by analyzing a network model of signaling pathways affected by psychological stress. These results provide insights into the mechanisms of psychological stress signaling in therapy-resistant cancer, and indicate the potential benefit of reducing psychological stress in designing more effective therapies for prostate cancer patients.

## Author Summary

Psychological stress and anxiety are often experienced by prostate cancer patients, but the underlying mechanisms of interactions between psychological stress and cancer development, as well as drug resistance, are unclear. Here, we employed a systems biology approach to study interactions between stress-activated epinephrine/beta2 adrenergic receptor/protein kinase A signaling and a regulatory network that controls apoptosis in prostate cancer cells. We developed a dynamic network model of signaling pathways that control apoptosis in prostate cancer cells and quantitatively evaluated the effects of stress-activated signaling on apoptosis induced by drug combinations. Experimental data were used to guide modeling, to fit the unknown parameters and validate the model. Based on our model we found that epinephrine/beta2 adrenergic receptor/protein kinase A signaling can decrease drug efficiency, and can shift the effect of drug combination from synergy to antagonism. We also predicted that in addition to BAD phosphorylation Mcl-1 expression could be upregulated by stress/epinephrine signaling to inhibit apoptosis. This study provides insights into the mechanisms of psychological stress signaling in therapy-resistant cancer, and suggests that reducing psychological stress could help to make prostate cancer treatment more effective.

## Introduction

Psychological stress has been implicated in cancer for almost 2 millennia. It has been observed that psychological stress may contribute to cancer initiation and progression [1], [2]. However, the causal relationship between stress and cancer remains poorly understood [3], largely because of limited information about how stress could influence tumor development and drug resistance [4], [5].

Our recent experiments in an animal model [5] demonstrated that injections of epinephrine or immobilization stress counteracted the anti-tumor effects of PI3K inhibitors on prostate cancer xenografts in mice. Based on these observations, we hypothesized that psychological stress activates anti-apoptotic signaling in prostate cancer cells and, as a result, contributes to the progression of prostate cancer and chemotherapeutic resistance in advanced prostate cancer. Our experiments [5]– have demonstrated that tumor-promoting effects of stress depend on phosphorylation of BAD, a member of the BH-3 only subfamily of Bcl2 proteins. BAD is phosphorylated at Ser^112^ through the epinephrine-beta2 adrenergic receptor (β2AR)-PKA-BAD anti-apoptotic signaling pathway [5]–[7]. BAD can also be phosphorylated by other signaling pathways. For example, epidermal growth factor (EGF) triggers phosphorylation of BAD at Ser^112^ through the EGFR-Raf-MEK/ERK-KinaseX pathway and at Ser^136^ through the Rac-PAK pathway [8], whereas activated PI3K transmits signals to Ser^136^ through AKT activation, and also regulates Ser^112^ via an unidentified mechanism partially dependent on Akt [8].

To extend analysis of interactions between stress and apoptosis beyond single linear pathway, we used a systems biology approach to study interactions between stress-activated signaling and a regulatory network that controls apoptosis in prostate cancer cells.

Several mathematical models of apoptosis regulation have been developed. A Boolean model of apoptosis [9] was proposed to qualitatively analyze the central intrinsic and extrinsic apoptosis pathways and connected pathways. Continuous modeling based on kinetic laws, such as the law of mass action and Michaelis-Menten kinetics, is an alternative approach. Constituted by differential equations, a model of the signaling pathways governing apoptosis [10] demonstrated that inhibition of caspase 3 and caspase 9 resulted in an implicit positive feedback and in bistability. Recently, a mathematic model of Src control on the mitochondrial pathway of apoptosis [11] was designed and fitted to experimental data, used for theoretical design of optimal therapeutic strategies.

However, no models have examined interactions between signaling activated by psychological stress, apoptosis, and drug resistance, particularly, resistance to drug combination therapy [12]–[14]. We developed a systems biology model to examine the role of psychological stress in apoptosis regulation and therapeutic sensitivity, and to further analyze the associated signaling pathways activated by stress hormones. By comparing predictive power of two different models with or without the role of Mcl-1, we predicted that in addition to BAD phosphorylation Mcl-1 expression could be upregulated by stress/epinephrine signaling to inhibit apoptosis. Overall our modeling showed that stress/epinephrine signaling interfered with apoptosis induced in prostate cancer cells by combinations of signal transduction inhibitors.

## Results

### Experiment-guided mathematical modeling of stress-mediated anti-apoptosis pathways

**Figure 1:**
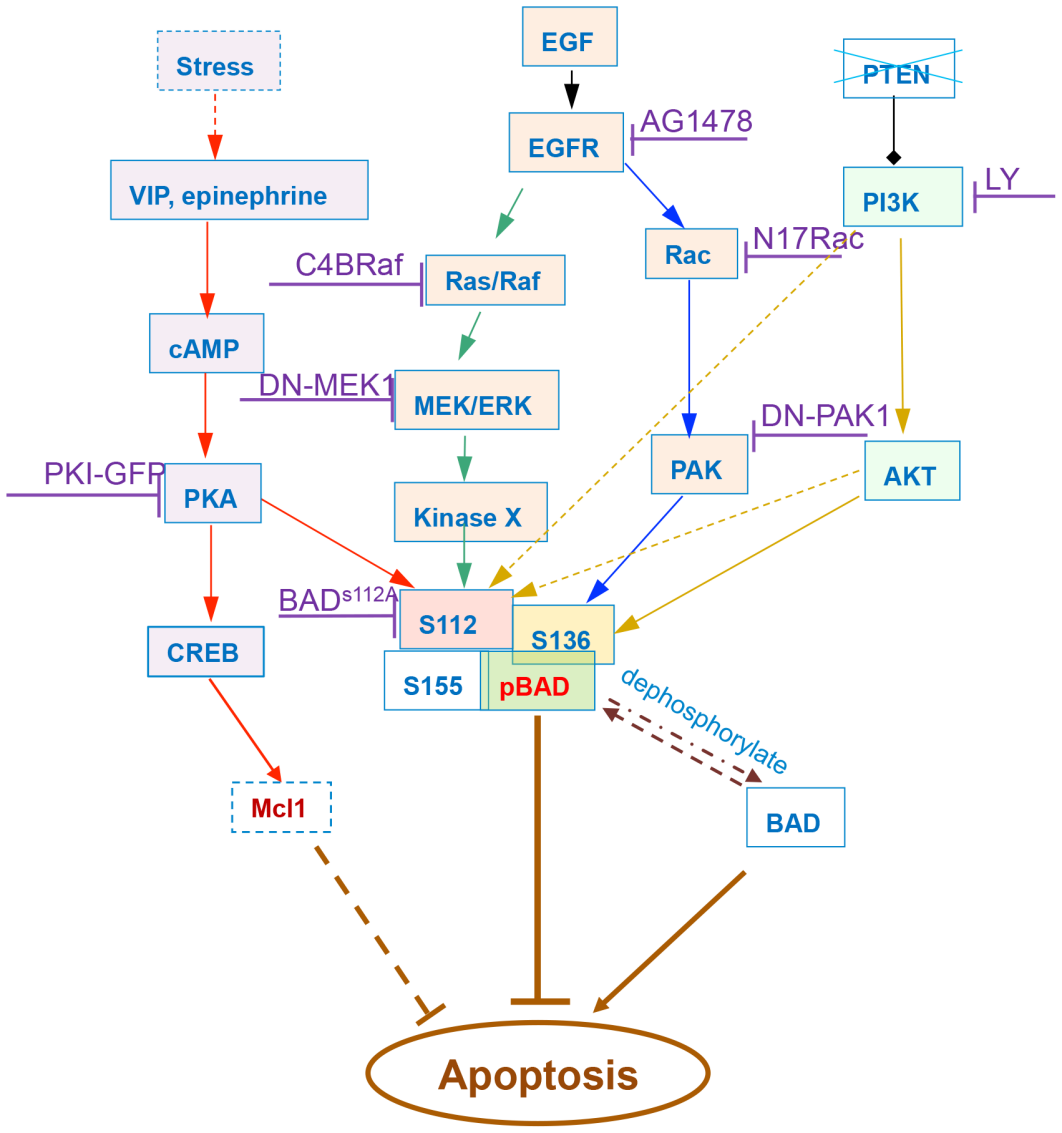
BAD signaling pathway of apoptosis regulation in prostate cancer cells, mediated by psychological stress. Phosphorylated BAD is critical for the anti-apoptotic effect of multiple signaling pathways, while dephosphorylated BAD has a pro-apoptotic effect. Stress, EGF, and PI3K can activate independent signaling pathways that phosphorylate BAD. Stress, represented by epinephrine (or VIP) can promote the phosphorylation of BAD at Ser^112^ via activation of cAMP and PKA. EGF phosphorylates BAD at Ser^112^ through the Ras/Raf-MEK/ERK-KinaseX pathway and at Ser^136^ through the Rac-PAK pathway. PI3K transmits signals to Ser^136^ through AKT activation, and AKT can partially regulate Ser^112^. The anti-apoptotic role of the activation of CREB and Mcl-1 induced by stress was determined by comparing the predictive power of different models. Multiple drugs targeting different signaling pathways were integrated into the model.

BAD is a convergence point for several anti-apoptotic signaling pathways in prostate cancer cells. Phosphorylated BAD is critical for the anti-apoptotic effects of such signaling pathways, while dephosphorylated BAD has pro-apoptotic effects. Stress, EGF and PI3K can activate independent signaling pathways that phosphorylate BAD (**Figure 1**). These signaling pathways form a convergent network that control apoptosis via BAD phosphorylation.

We modeled these signal transduction networks using a system of ordinary differential equations (ODEs) to describe the dynamic phosphorylation and dephosphorylation of each protein in the pathways. The model was built according to Michaelis-Menten kinetics [15] using Hill functions [16], [17].

**Figure 2:**
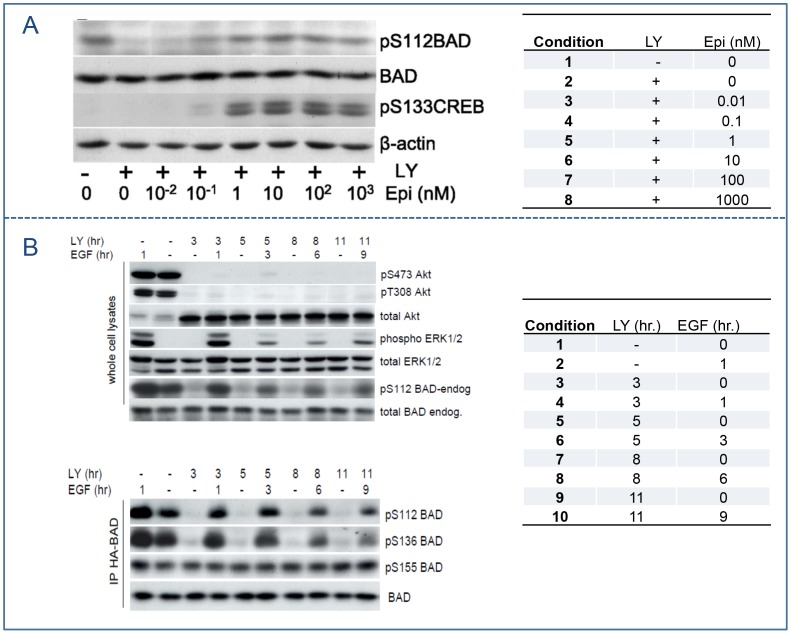
Western blots for protein phosphorylation in stress-mediated BAD signaling pathway. Both experiments were conducted in LNCaP cells. (A) Protein phosphorylation in cells treated with 50 µm LY294002 for 2 hours followed with increasing concentrations of epinephrine (0.01–1000 nm) for 1 h. BAD phosphorylation at Ser112 and CREB phosphorylated at Ser133 were measured. (B) Protein phosphorylation in cells treated with LY294002 (LY) followed with EGF 2 h later. Phospho-Ser473 Akt, phospho-Thr308 Akt, total Akt, phospho-ERK1/2, total ERK1/2, phospho-Ser112 BAD, and total BAD were measured for the indicated times. LY294002 inducing dephosphorylation of HA-BAD at Ser112 and Ser136 were also followed by Western blot analysis. Data from [6], [7]. Panel A reproduced from [7] and Panel B reproduced from [6] with permission from the American Society for Biochemistry and Molecular Biology.

Our experimental data (**Figure 2**) demonstrated that the phosphorylation of ERK1/2 peaks under the stimulation of EGF, and then decreases within 1 hour due to the short term signaling of the epidermal growth factor receptor (EGFR) [18]. Therefore, we described the de-phosphorylation rates of each protein in the EGFR-Ras-ERK1/2-KinaseX pathway to be dependent on both its phosphorylation and dephosphorylation level and time course as in Equations (1–4) below,
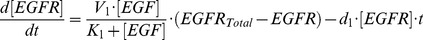
(1)

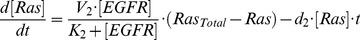
(2)

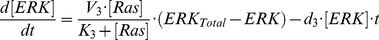
(3)

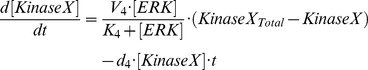
(4)Where 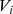 and 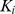 are maximal activation velocities and Michaelis activation coefficient of each protein by its upstream regulator, respectively. By multiplying the constant dephosphorylation coefficient 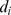 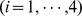 with time *t*, Equations (1–4) can reproduce the signaling curves with peaks followed by later declines.

**Figure 3:**
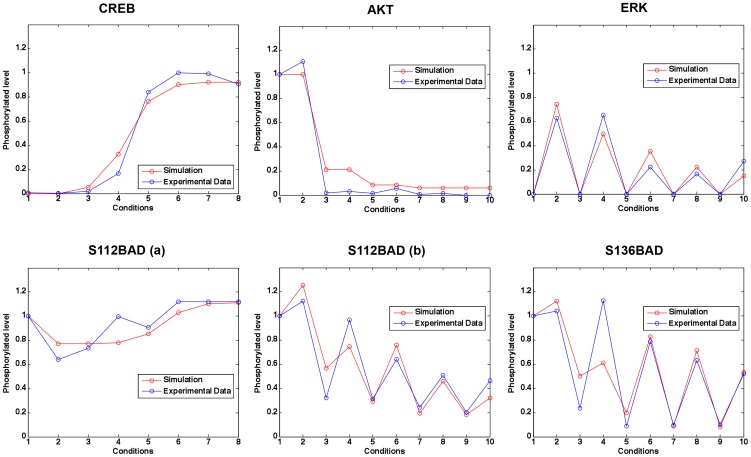
Simulation results of phosphorylated levels of some proteins under different conditions compared to experimental data. Different conditions correspond to different treatments (see right panel of Figure 2). Mean squared error between the simulated data and experimental data is 0.1211.

The other signaling regulations regarding phosphorylation or activation of Rac, PAK, PI3K, AKT, PKA, cAMP, PKA, CREB, S112BAD and S136BAD were also modeled by ODEs using Hill functions as described below in Equations (5–13), where the dephosphorylation rates were modeled as constants calculated by ensuring the existence of the steady states of these proteins (see Materials and Methods).
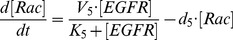
(5)

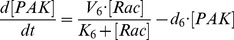
(6)

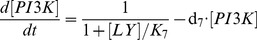
(7)

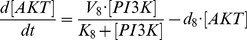
(8)

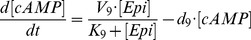
(9)

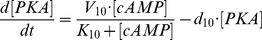
(10)

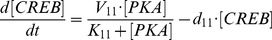
(11)

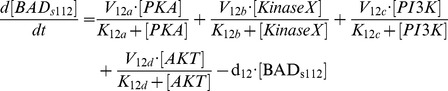
(12)

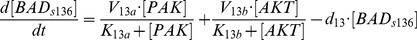
(13)We then fitted unknown parameters in the model to the experimental data (see Materials and Methods). The estimated parameter values involved in the modeled signaling pathways are listed in **Table S1**. **Figure 3** shows that the simulations are consistent with the experimental data (mean squared error between the simulated and experimental data = 0.1211).

**Figure 4:**
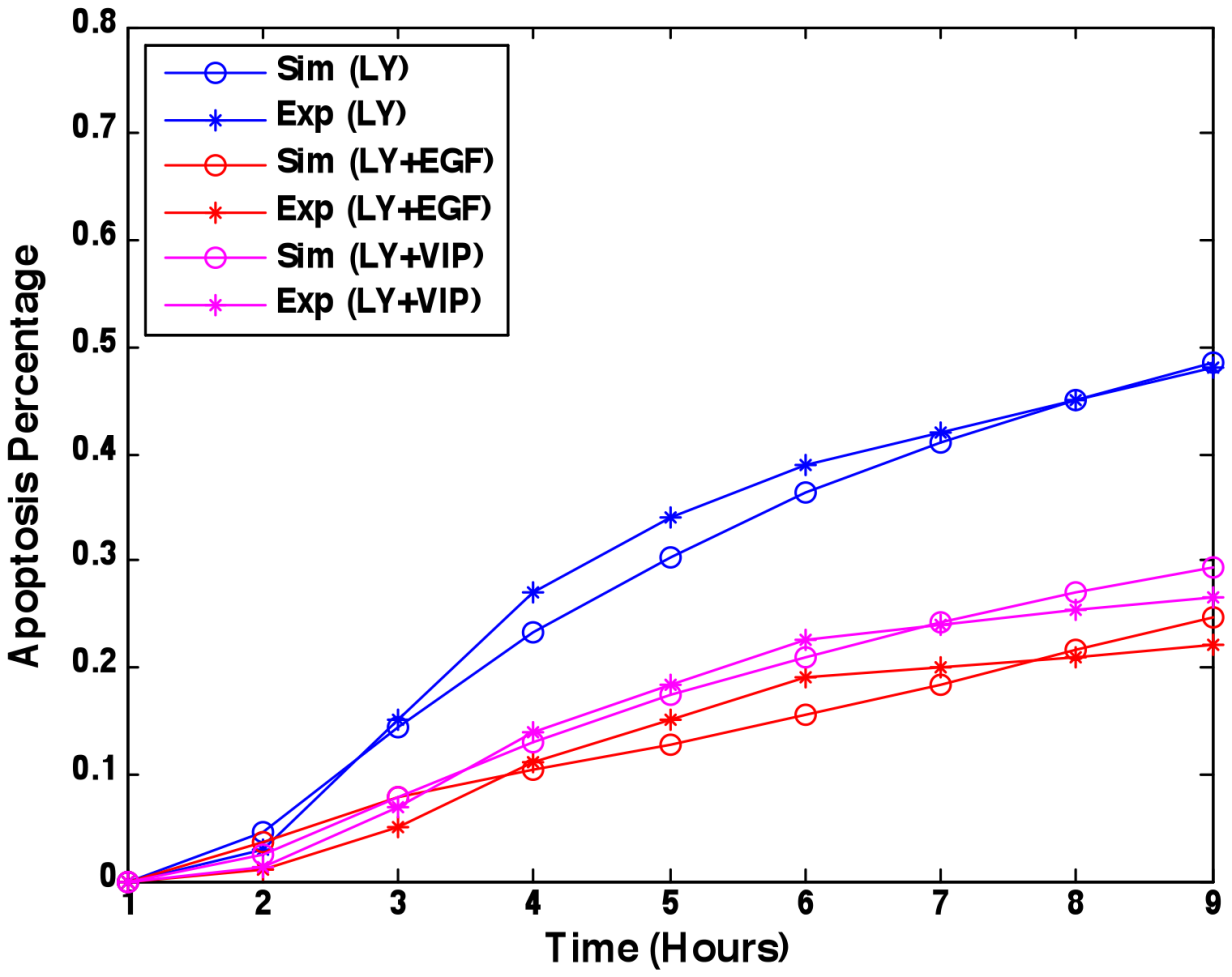
Apoptosis percentage prediction. Parameters were fitted to experimental data [6], [8] under the treatments of LY294002, LY294002 & EGF, and LY294002 & VIP. Mean squared error between the simulations and experimental data is 0.0221.

Next we linked the BAD phosphorylation signaling pathways established above to apoptosis percentage. Recently, the preliminary experimental study in our lab indicated that, besides BAD, Mcl-1 may be also involved in stress-mediated apoptosis regulation [19], [20]. Thus, we considered one model based on BAD phosphorylation only (see Equation 14.1 below) and one based on both BAD phosphorylation and stabilization of Mcl-1 (see Equation 14.2 which accounts for the potential role of stress-induced activation of CREB, leading to increased transcription of Mcl-1 independent of BAD phosphorylation).
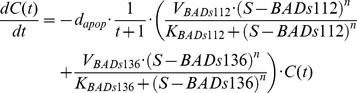
(14.1)

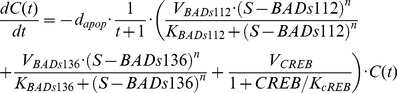
(14.2)

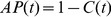
(15)where 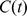 is cell survival percentage and 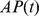 is apoptosis percentage. 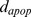 is the apoptosis rate in prostate cancer cells. 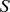 represents total BAD. The additive incorporation of 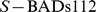 and 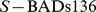 in Equation (14) implies that phosphorylation at either S112 or S136 is sufficient to inhibit pro-apoptotic function of BAD, as previously observed [6], [21]. The potential role of Mcl-1 will be verified by examining its predictive power. The unknown parameters in Hill functions including 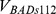, 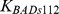, 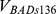, 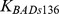, 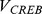, 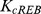, 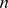, and apoptosis rate, 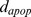, were fitted to our experimental data (**Figure S1**) by a procedure similar to that above (see Materials and Methods); estimated values are listed in **Table S2**. We did not explicitly model the regulation of apoptosis by some proteins or transcription factors (e.g. BclXL, BAX and BAK [22]) involved downstream of our considered pathways. In an implicit approach, indicated by fitting to the evolution of experimental apoptosis percentage (**Figure S1**), we modeled the time-dependent nonlinear regulation of apoptosis by multiplying 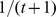 to the right hand of the equation, which resulted in a better data fit. **Figure 4** shows prediction of apoptosis percentage in the model with Mcl-1 compared to the experimental data (mean squared error = 0.0221).

**Figure 5:**
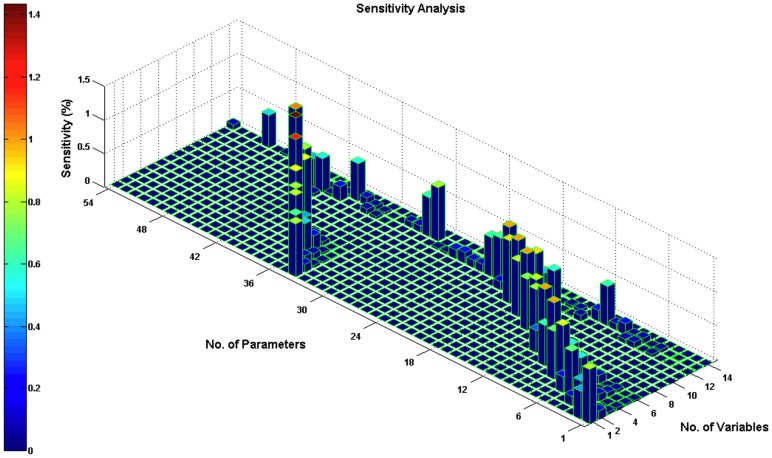
Sensitivity analysis for the estimated parameters. Variables 1–13 correspond to the proteins in Equation (1–13) respectively; variable 14 is apoptosis percentage. Parameters 1–54 were listed in Table S1, 2. Each parameter was increased by 1% from its estimated value; then we obtained the time-averaged percentage change of each variable value. All the sensitivity values were below 1.4327%. The sensitivity analysis result confirmed that the developed system is preserved to the modest parameter changes and our model is rather robust.

Here, we theoretically analyzed the stability of the developed system. Let 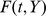 denote the vector of functions in the right hand of the Equations (1–15) with 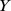 the vector of proteins phosphorylation considered. Then 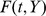 is Lipschitz continuous with respect to 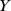 uniformly in the range 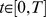 for any finite *T*, therefore the developed system continuously depends on the initial values and parameters [23]. We then performed a sensitivity analysis for the estimated parameters (see Materials and Methods). Each parameter was increased by 1% from its estimated value, and then we obtained the time-averaged percentage change of each variable value. All sensitivity values were not more than 1.4327% (**Figure 5**). The sensitivity analysis result confirmed that the developed system is conserved through the modest parameter changes and our model is rather robust.

### Integrating effects of inhibitor interventions targeting signaling pathways

Currently several inhibitors that target the BAD upstream signaling network are in clinical trials, including PI3K inhibitors (e.g. LY294002 (LY), CAL-101, BKM120, and GDC-0941), EGFR inhibitors (e.g. gefitinib, erlotinib HCl) and MEK inhibitors (e.g. AZD6244, GSK1120212). As shown in **Figure 1** we considered 8 pharmacological and dominant negative inhibitors of signaling downstream of the EGF, PI3K and psychological stress pathways: the PI3K inhibitor LY294002, the EGFR tyrosine kinase inhibitor AG1478, the Rac inhibitor N17Rac, the PAK inhibitor DN-PAK1, the RAF inhibitor C4BRaf, the MEK/ERK1/2 inhibitor DN-MEK1, the PKA inhibitor PKI-GFP, and BADS112A as a functional equivalent of an inhibitor of BAD phosphorylation at S112 [5], [6].

The inhibition effect of LY294002 (LY) was modeled in Equation (7) using an inhibition Hill function. Inhibition effects of the other inhibitors were also modeled by multiplying an inhibition Hill function to the maximal reaction velocity (see Equations 1–3, 5, 6, and 10, respectively). We integrated these inhibition effects by redefining each 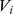 as:
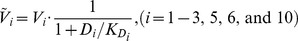
(16)where 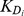 is the Michaelis-Menten constant indicating the concentration of drug 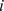 that decreases the maximal reaction velocity 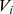 to half the original value without drug treatment. In this work we normalized the concentration of drug 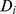 to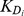. As a result, the non-dimensional value of the drug concentration became 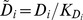. Thus, we did not introduce any additional parameters into the model.

Mutant BADS112A inhibits the anti-apoptotic role of phosphorylated pS112BAD by decreasing the relative ratio of phosphorylated pS112BAD to dephosphorylated S112BAD that binds BclXL and promotes apoptosis. Therefore, we assumed that BADS112A decreases the relative level of steady phosphorylation of S112BAD, which was modeled by integrating drug effects into the dephosphorylation rate of S112BAD as follows,
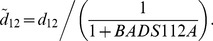
(17)


### Experimental validation and model selection revealing the anti-apoptotic role of Mcl-1

To investigate the potential role of Mcl-1 transcription in anti-apoptosis, we compared two different models of anti-apoptosis regulation: one based on BAD phosphorylation only, and one based on both BAD phosphorylation and stabilization of Mcl-1, as modeled in Equations 14.1 and 14.2, respectively.

**Figure 6:**
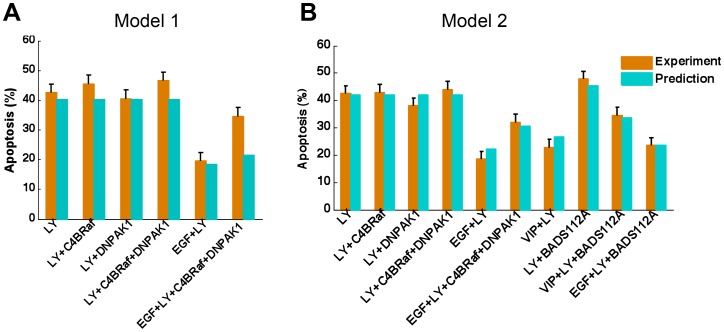
Experimental validation and model selection. (A) Model 1: Apoptosis regulated by BAD phosphorylation only. (B) Model 2: Apoptosis regulated by both BAD phosphorylation and stabilization of Mcl-1. The predicted apoptosis percentages under various treatments or conditions were compared to the experimental data [6], [8]. Single LY294002 and its combinations with other inhibitors (C4BRaf, DNPAK1 and BADS112A) or growth factors/cytokines (EGF and VIP) were used for validation. Model 2 shows better predictive power than model 1.

The predictions of apoptosis percentage under different treatments or conditions were compared to the experimental data [6], [8] (**Figure S1**). Experimental data were normalized to the same experimental environment. The prediction of apoptosis percentage for EGF&LY&C4BRaf&DNPAK1 in the first model (**Figure 6A**) was not consistent with the experimental data. The second model (**Figure 6B**) improved validation and presented better predictive power, and emphasized the potential role of Mcl-1 in anti-apoptotic effects of emotional stress/epinephrine.

Our selected model, the second model with Mcl-1, predicted that LY294002, LY294002 & C4BRaf, LY294002 & DNPAK1, LY294002 & DNPAK1 & C4BRaf should have similar effects on the percent apoptosis of cancer cells, which was consistent with the experimental data (**Figure 6B**). The prediction that LY294002 plus BADS112A would produce the best pro-apoptotic effect was experimentally validated. Moreover, addition of EGF or activation of PKA signaling by epinephrine inhibited apoptosis induced by a single inhibitor or a combination, shown both in the model and experimentally. The agreement between the predicted and the experimental results confirmed that our model can quantitatively predict apoptosis percentage of prostate cancer cells under various treatments and different conditions.

### Quantitative evaluation of inhibitor combination

**Figure 7:**
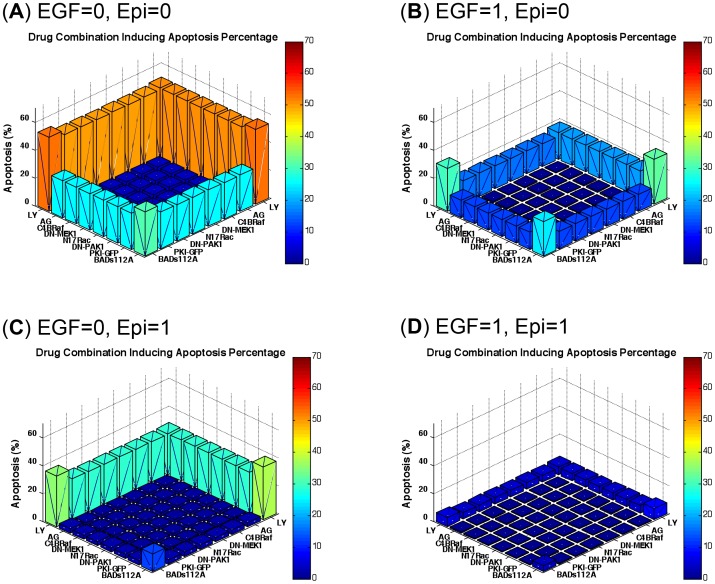
Drug combination prediction with or without EGF and/or epinephrine. (A) Without EGF and epinephrine. (B) With EGF but no epinephrine. (C) With epinephrine but no EGF. (D) With both EGF and epinephrine. The combination of LY294002 and BADS112A has the greatest effect among all combinations of 8 inhibitors under all conditions examined. EGF and epinephrine (stress) reduced drug efficiency.

Then we investigated the effects of combined signaling inhibitors on apoptosis percentage with or without EGF and/or epinephrine. Since the combination of more than 3 drugs is less realistic for clinical purposes and may lead to unknown side effects, we limited our considerations to a combination of two inhibitors. The dose of each inhibitor in the pairs was set as 1, so the total dose of each combination was 2, which was the same for one single inhibitor “combined” with this inhibitor itself. **Figure 7A** shows the apoptosis percentages induced by inhibitor combinations under conditions without EGF and epinephrine. The signaling pathways stimulated by EGF and psychological stress were inactivated and the apoptosis percentage was effectively promoted by all inhibitors. LY294002 showed a strong pro-apoptotic effect as a single treatment or combined with other inhibitors, and BADS112A had less effect. **Figure 7B** shows the combinatorial effects of inhibitors with EGF but no psychological stress. The apoptosis percentages were decreased compared to **Figure 6A**. However, LY294002 combined with BADS112A demonstrated a much stronger pro-apoptotic effect compared to other combinations. **Figure 7C** shows the effects of inhibitor combinations plus epinephrine. Pro-apoptotic effects of all combinations of inhibitors, except for BADS112A with LY294002, were inhibited by stress-activated signaling. Finally, when both EGF and epinephrine were present, pro-apoptotic effects of all inhibitor combinations were substantially decreased (**Figure 7D**). These results demonstrate variability of apoptosis induction by different combinations of inhibitors, in the presence of agents that activate anti-apoptotic pathways.

Based on our modeling, the combination of BADS112A and LY294002 produces the greatest effect on promoting apoptosis in prostate cancer cells. Therefore, we tested whether this combination of BADS112A and LY294002 is synergistic [24], [25]. We first adopted the Loewe additivity [26]–[28] to quantitatively evaluate and examine the synergism of LY294002 plus BADS112A.

The Loewe combination index is defined as a ratio of total effective drug dose (combination versus single drug) required to achieve a given effect as follows:
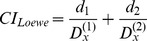
(18)where *d_1_* (BADS112A) and *d_2_* (LY) are the doses in the combination isobologram with respect to the 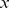 percentage of apoptotic cells. 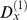 and 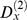 represent the concentration of BADS112A and LY294002 with respect to promoting apoptotic cells by 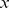 percentage, respectively. *CI_Loewe_*<1, *CI_Loewe_*>1 and *CI_Loewe_* = 1 indicate Loewe synergy, antagonism, and additivity, respectively.

**Figure 8:**
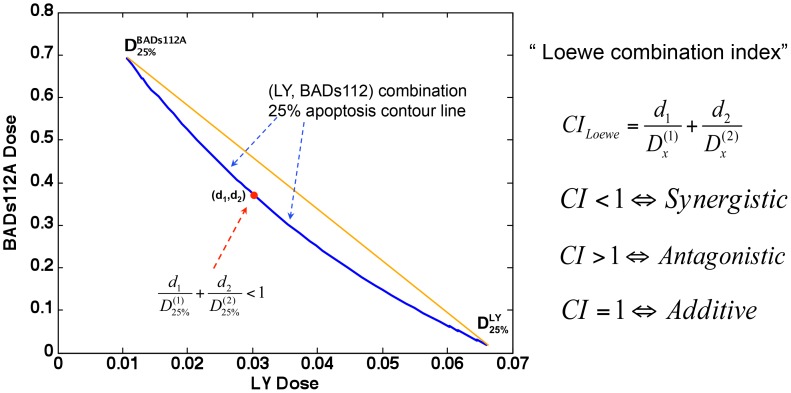
Synergy prediction of LY294002 and BADS112A combination based on 25% apoptosis isobologram. Loewe Index was used to evaluate the combinatorial effect of LY294002 and BADS112A. 25% isobologram of BADS112A and LY294002 (blue curve) bows inward, indicating *CI_Loewe_*<1. The combination of BADS112A and LY294002 is synergistic regarding the 25% percent apoptosis isobologram.

**Figure 8** shows that 25% isobologram of BADS112A and LY294002 (blue curve) bows inward, indicating *CI_Loewe_*<1. Therefore the combination of BADS112A and LY294002 is synergistic regarding the 25% apoptosis isobologram.

To calculate the Loewe index requires solving a reverse problem based on an isobologram. Thus, this approach requires a high computing cost and consideration for specific isobolograms. Another quantification method for combination therapies is Bliss independence [27], [29]. But the calculation of this qualification index resulted in negative expected apoptosis percentage values of combined inhibitors, which is not realistic. Thus, indicated by (but different from) the Bliss index, we defined a new combination index as follows:
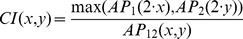
(19)where 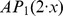 is apoptosis percentage induced by 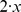 doses of inhibitor 1, and 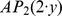 is apoptosis percentage induced by 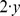 doses of inhibitor 2. 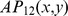 is the apoptosis percentage promoted by combined inhibitor 1 and inhibitor 2 with 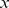 dose and 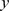 dose, respectively. With the same total doses, if the combined inhibitors produce a greater effect than both single inhibitor 1 and inhibitor 2, the index considers that these two inhibitors work synergistically. Therefore, the index considers the combination as a synergism effect if CI <1, as antagonism if CI>1, and otherwise additivity.

### Synergism switch induced by psychological stress

**Figure 9:**
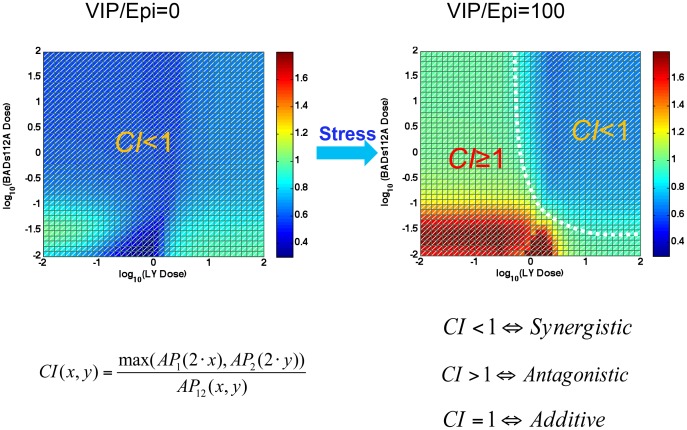
Synergism pattern switch triggered by psychological stress. In the no or low psychological stress environment (i.e. VIP or epinephrine set close to 0 in the simulation), the index was less than 1, which indicated that BADS112A plus LY294002 has synergistic effect in the whole dose region we considered. Whereas as high psychological stress emerged (VIP or epinephrine set as 100 in the simulation), the synergism switched to a different pattern that was divided into two regions: one synergism (CI<1) in high dose region and another antagonism or additivity (CI> = 1) elsewhere.

We evaluated dose-dependent synergism of combined BADS112A and LY294002 as defined in Equation (19) with or without psychological stress. In the simulation, the dose of each inhibitor ranged from 0.01 to 100. In the no or low psychological stress environment, BADS112A plus LY294002 has a synergistic effect, but in the high psychological stress environment, the synergism pattern switched (**Figure 9**). The synergism pattern was divided into two regions: one with CI<1 indicating synergism and another with CI≥1 corresponding to antagonism or additivity. Therefore, psychological stress triggered the synergism pattern switch to a dose-dependent combination synergism. Under the high psychological stress condition, only if the doses of BADS112A and LY294002 were high enough, did their combination produce synergism with respect to promoting apoptosis of cancer cells.

Stress could decrease the efficiency of anti-cancer therapy (**Figure 1**). A dose-dependent response of BADS112A and LY294002 combination therapy in **Figure S2** further demonstrates drug resistance induced by psychological stress. When the stress (or epinephrine) was absent, the apoptosis percentage was slightly affected by the doses of LY294002 and BADS112A and remained at a high level. While when the psychological stress emerged, high doses and low doses of LY294002 resulted in different levels of apoptosis percentage, even when combined with the high doses of BADS112A. The drug resistance induced by stress was consistent with the switch of synergism pattern as demonstrated above.

**Figure 10:**
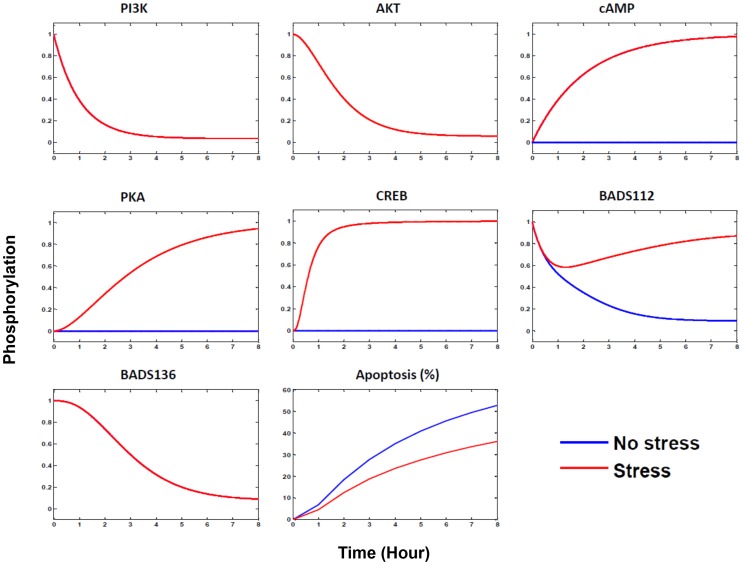
Molecular responses to the BADS112A and LY294002 combination therapy with (red) or without (blue) psychological stress. The time course ranges from 0 to 8-ADRB2-cAMP-PKA transducing to both S112BAD and CREB (or Mcl1), stimulated by psychological stress are responsible for the drug resistance and synergism pattern switch in drug combination therapy. Psychological stress activated epinephrine-ADRB2-cAMP-PKA signaling pathway, which counteracted the repression of S112BAD by proposed inhibitors and decreased their apoptosis-inducing effects. Independently of S112BAD and S136BAD, the CREB (or Mcl-1) pathway activated by PKA added an anti-apoptotic role.

We then examined the differences in signaling pathways with or without psychological stress with combination therapy. When there was no psychological stress, the epinephrine-β2AR-cAMP-PKA-CREB signaling pathway was not activated. PI3K-AKT pathway was inhibited by LY294002, and the relative phosphorylation of BAD at S112 and S136 was repressed to a low level around 0.1 (**Figure 10**). When psychological stress was introduced, the epinephrine-β2AR-cAMP-PKA signaling pathway was activated leading to phosphorylation of BAD at S112, which counteracted the repression of BAD phosphorylation at S112 induced by LY294002 and BADS112A. As a result, the relative phosphorylation of BAD at S112 returned to a higher level.

In addition to phosphorylation of BAD at S112 and s136, Mcl1 (or CREB) activated by stress signaling could also inhibit apoptotic, so percent apoptosis in cancer cells was decreased compared to the no-stress condition (**Figure 10**). Therefore, the differentially activated signaling pathways stimulated by psychological stress, leading to both BAD phosphorylation and Mcl-1 activation, were responsible for the drug resistance and synergism pattern switch in combination therapy.

## Discussion

Our modeling strategy successfully captured key kinetic features of the underlying signaling pathways discussed above. We did not describe the kinetics in the pathway by linear equation based on mass action law, since the detailed reaction was unclear and ignorable. Instead, we incorporated by Michaelis-Menten kinetics using the Hill function [16], [17] to integrate less critical reaction details. Based on experimental data, we phenomenologically modeled the rate of change for dephosphorylation of proteins in EGFR-ERK1/2 pathway and apoptosis regulation to be time dependent. The simulation results (**Figure 3**) were consistent with experimental data (**Figure 2**), which suggested the fundamental signaling networks used in this work were reliable. In future work, we will integrate elements downstream of BAD, such as BclXL, BAX and BAK [22], to investigate a more detailed mechanism related to stress interactions in prostate cancer.

The anti-apoptotic role of BAD phosphorylation mediated by emotional stress has been well studied. Recently, our lab found that, besides BAD, Mcl-1 may be also involved in stress-mediated apoptosis regulation (Hassan et al unpublished data). Here, we applied a systems biology approach to investigate the potential role of Mcl-1 stabilization in anti-apoptotic effects of emotional stress/epinephrine, which was verified by comparing the predictive power of two different models with or without the role of Mcl-1. The selected model with better predictive power will be used to explore effects of stress on Mcl-1 in our ongoing experiments.

Effects of drugs on apoptosis varied depending on which components in the signaling network were targeted. This was due to kinetic asymmetry of different signaling pathways. As shown in **Figure 1**, PI3K/AKT pathway can phosphorylate BAD at both S112 and S136 [8], so the inhibition of this pathway by PI3K inhibitor LY294002 could induce more cell death compared to other inhibitors, such as N17Rac or DN-PAK1, that target pathways that phosphorylate only one site of BAD. Expression of phosphorylation-deficient mutant BADS112A can also effectively promote apoptosis [5], [6]. Finally, drug-induced signaling network remodeling is an important and interesting question for future work.

Psychological stress and anxiety are often experienced by prostate cancer patients. The increased psychological stress that can result from cancer progression and diagnosis strengthens the activation of anti-apoptotic signaling pathways [19], as demonstrated in our simulation, which could decrease therapy efficiency and shift drug combinations from synergy to antagonism. These results also suggest the need for deeper analysis of the role of stress-related signaling in other therapy-resistant cancers.

In summary, we developed a dynamic network model of signaling pathways that control apoptosis in prostate cancer cells to study the role of psychological stress on prostate cancer therapy, and justified the role of Mcl-1 stabilization in anti-apoptotic effects of emotional stress. A drug resistance and synergism switch was revealed in our model, and the associated signaling mechanisms were explored.

## Materials and Methods

### Experimental data

We collected data at both the molecular and cellular levels. The molecular data regarding protein phosphorylation included two sets of Western blotting images [6], [7], both done in LNCaP cells. In the first experiment (**Figure 2A**), cells were treated with 50 µm LY294002 for 2 hours followed with increasing concentrations of epinephrine (0.01–1000 nm) for 1 h. BAD phosphorylation at Ser112 and CREB phosphorylated at Ser133 were measured. The second set of data (**Figure 2B**) contains the time course of protein phosphorylation in cells treated with LY294002 followed with EGF 2 hours later. Phospho-Ser473 Akt, phospho-Thr308 Akt, total Akt, phospho-ERK1/2, total ERK1/2, phospho-Ser112 BAD, and total BAD were measured for the indicated times. LY294002, inducing dephosphorylation of HA-BAD at Ser112 and Ser136, was followed by Western blot analysis (**Figure 2B**). We quantified the Western blotting data using ImageJ software and the normalized values were listed in **Table S3**. For the first experimental data set, there were 8 conditions with or without LY294002 treatment and with increasing concentrations of epinephrine. The concentration of phosphorylated BADs112 was normalized to the control condition without LY294002 treatment and epinephrine. Phosphorylated CREB was normalized to the maximal concentration, since the concentration in the control condition was minimal. For the second experimental data set, there were 10 treatment conditions with different time periods of LY294002 and EGF treatment. Concentrations of phosphorylated S473Akt, S112BAD, and S136 BAD were normalized to the control condition (neither LY294002 nor EGF treatment). Phosphorylated ERK1/2 concentration was normalized to total ERK1/2 concentration, since the concentration of phosphorylated ERK1/2 under the control condition was almost zero. These data were used to estimate the parameters in Equations (1–13).

The cellular level data from [6], [8] were apoptosis percentages determined by counting at least 350 cells in several randomly chosen fields for every treatment. Considering that the experimental data were conducted in different experimental environments, we scaled the data in **Figure S1B, C** to the data in **Figure S1A** to ensure the apoptosis percentages under the treatments of LY294002 and LY&EGF were at the same levels. Treatments of LY, LY&EGF, and LY&VIP were used to estimate the parameters in Equation (14); the remaining data were used to validate model predictions.

We measured apoptosis by several independent methods: 1) caspase assay – a quantitative assay that measures activity of effector caspase 3 against fluorogenic substrate DEVD-amc [6]; 2) time-lapse video microscopy- a quantitative assay that follows morphological changes of individual cells over 24 hours [6]; 3) western blotting for apoptosis markers– cleaved caspase 3, caspase 7 and cleaved PARP, this is qualitative assays that confirms activation of caspases and cleavage of physiological substrate in dying cells [30]; 4) immunofluorescent staining for active caspase 3 and release of cytochrome c from mitochondria– a specific hallmark of apoptosis [30], [31]; 5) TUNEL assay, this assay detects cleaved DNA – specific hallmark of apoptotic cell death [20]. Of these methods caspase assay and time lapse video microscopy are considered most appropriate to quantitatively measure apoptosis. Other methods confirm that cell death is indeed by apoptosis mechanism. These methodologies are consistent with published “Guidelines for the use and interpretation of assays for monitoring cell death in higher eukaryotes” [32].

### Parameter estimation

We estimated the unknown parameters in the model by fitting the simulation results to the experimental data described above. Equation (20) was employed for parameter estimation by minimizing the fitness error between the experimental and simulated data,
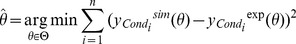
(20)where 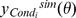 and 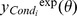 represent the simulated and experimental data with parameters 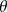 under condition 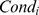
_,_ respectively. 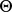 stands for the parameter space, in which the search space for each parameter was preset in a range according to the experimental observations and Michaelis-Menten kinetics.

According to the experimental data (**Figure 2**), we set the initial values of Equations (1–13) as the vector (0, 0, 0, 0, 0, 0, 1, 1, 0, 0, 0, 1, 1) in the simulation. To further reduce the numbers of unknown parameters, the parameters 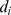, 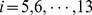, were calculated by ensuring the existence of the steady states of the system, for example, the dephosphorylation rate of PI3K, *d_8_*, was set as
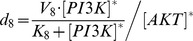
(21)where 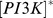 and 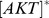 are steady states of PI3K and AKT which are assumed as 1 equal to their initial concentrations, respectively.

The remaining parameters, including 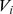, 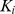
(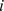 = 1, 2, …, 13a, 13b) and *d_1_, d_2_,…, d_4_*, were estimated using the above optimization procedure, for a total of 37 parameters in Equations (1–13) that were estimated by fitting to 56 experimental data points under different conditions. Similarly, 7 parameters in Equation (14) were estimated by fitting to 27 experimental data points.

A genetic algorithm [33] was adopted to minimize the cost function in Equation (20). The system of nonlinear ODEs was numerically solved using the 4^th^ Runge-Kutta method. The model simulation and result analysis were performed in MATLAB R2007b (MathWorks, USA).

### Sensitivity analysis

Parameter sensitivity analysis examines whether a system is preserved to the modest parameter changes and quantitatively explores the sensitive parameters. We used parameter sensitivity analysis to study the relationship between the proteins, apoptosis percentage and the variations for each parameter value. The relative sensitivity coefficient [34] of a variable 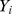 at time t with respect to a parameter 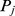 was computed by:
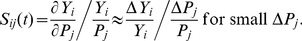
(22)Time-averaged sensitivities were calculated according to
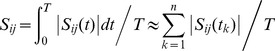
(23)where 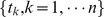 is an equal partition of 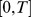. In the simulation, 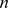 was set as 100 and 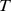 as 10. Each parameter was increased by a small perturbation, for instance 1%, from its estimated value, and then we obtained the time-averaged percentage change of each variable value.

## Supporting Information

Figure S1 **Experimental data of apoptosis percentage.** The percentages of apoptosis were determined by counting at least 350 cells in several randomly chosen fields for every treatment. The data from [6], [7] (for A, B) and [8] (for C) respectively Panel A reproduced from [6] with permission from the American Society for Biochemistry and Molecular Biology.
(TIF)

Figure S2 **A dose-dependent response of BADS112A and LY combination therapy induced by psychological stress.** When the stress (or epinephrine) was absent, the apoptosis percentage was slightly affected by the doses of LY and BADS112A and kept in a high level. While when the high psychological stress emerged, high dose and low dose of LY resulted in distinct apoptosis percentage even combined with the high doses of BADS112A.
(TIF)

Table S1 **Estimated parameters involved in the signaling pathway.**
(PDF)

Table S2 **Estimated parameters involved in apoptosis regulation.**
(PDF)

Table S3 **Quantification of experimental western blotting data.**
(PDF)
