# Supplementary figures and images for: Systems Modeling of Anti-apoptotic Pathways in Prostate Cancer: Psychological Stress Triggers a Synergism Pattern Switch in Drug Combination Therapy
Source: PLoS Comput Biol. 2013 Dec 5;9(12):e1003358. doi: 10.1371/journal.pcbi.1003358 (PMC3854132; doi:10.1371/journal.pcbi.1003358)

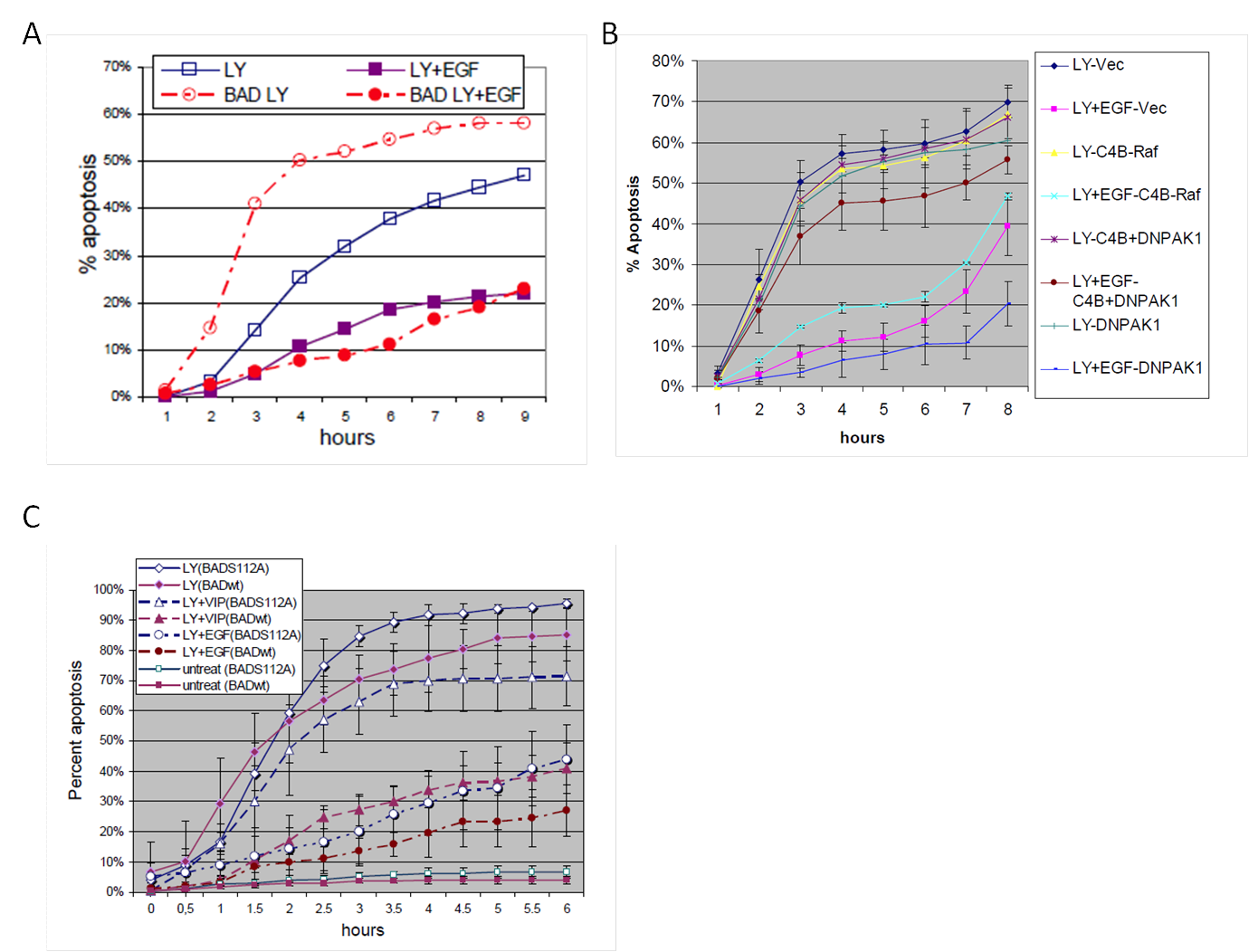

Supplement: Figure S1 — Experimental data of apoptosis percentage. The percentages of apoptosis were determined by counting at least 350 cells in several randomly chosen fields for every treatment. The data from [6], [7] (for A, B) and [8] (for C) respectively Panel A reproduced from [6] with permission from the American Society for Biochemistry and Molecular Biology. (TIF) [file pcbi.1003358.s001.tif]

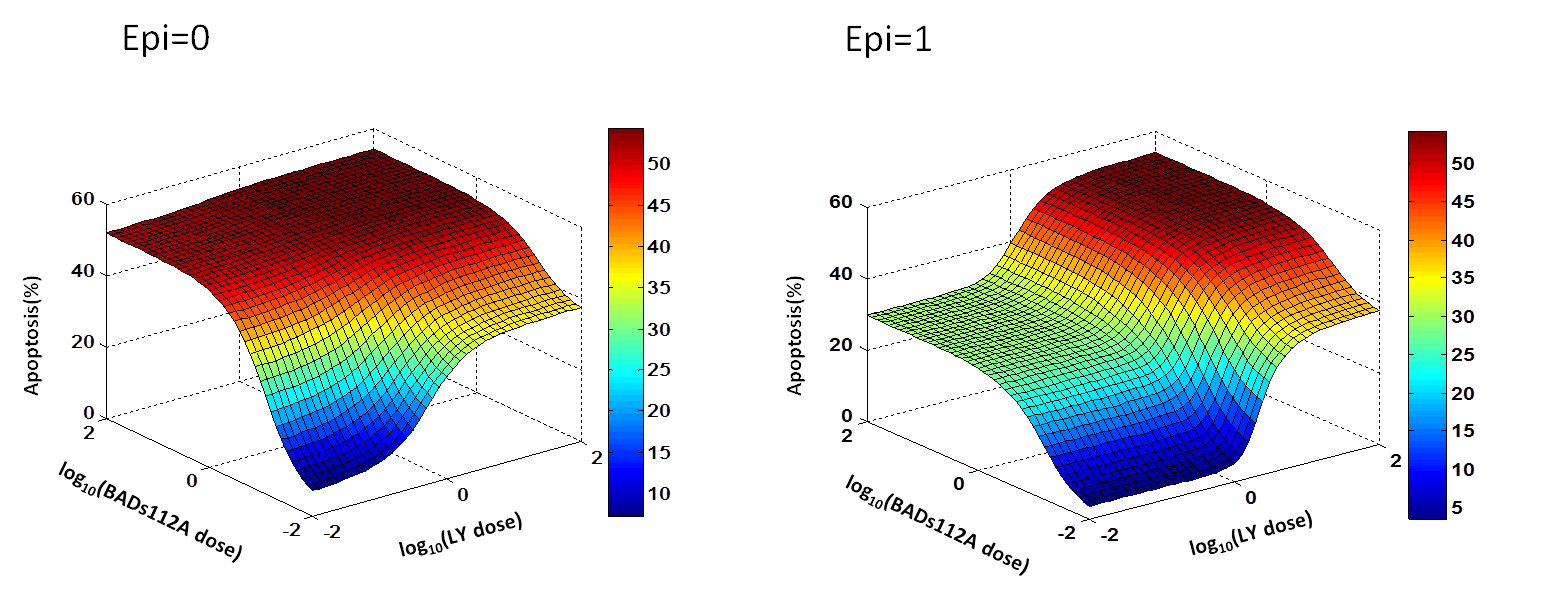

Supplement: Figure S2 — A dose-dependent response of BADS112A and LY combination therapy induced by psychological stress. When the stress (or epinephrine) was absent, the apoptosis percentage was slightly affected by the doses of LY and BADS112A and kept in a high level. While when the high psychological stress emerged, high dose and low dose of LY resulted in distinct apoptosis percentage even combined with the high doses of BADS112A. (TIF) [file pcbi.1003358.s002.tif]
