# Supplementary material for: Systems Modeling of Anti-apoptotic Pathways in Prostate Cancer: Psychological Stress Triggers a Synergism Pattern Switch in Drug Combination Therapy
Source: PLoS Comput Biol. 2013 Dec 5;9(12):e1003358. doi: 10.1371/journal.pcbi.1003358 (PMC3854132; doi:10.1371/journal.pcbi.1003358)
Supplement: Table S2 — Estimated parameters involved in apoptosis regulation. (PDF) [file pcbi.1003358.s004.pdf]

Para No.	Symbol	Value	Description
47	d_{apop}	0.3882	apoptosis rate of prostate cancer cells
48	$V_{BADs112}$	0.9961	Hill regulatory factor of apoptosis by S112BAD
49	$K_{BADs112}$	0.8235	Michealis constant in apoptosis regulation by S112BAD
50	$V_{BADs136}$	0.9765	Hill regulatory factor of apoptosis by S136BAD
51	$K_{BADs136}$	0.9922	Michealis constant in apoptosis regulation by S136BAD
52	V_{CREB}	0.1373	Hill regulatory factor of apoptosis by CREB
53	K_{CREB}	0.3608	Michealis constant in apoptosis regulation by CREB
54	n	5	Hill exponent of apoptosis regulation by BAD or CREB
