# Supplementary material for: Systems Modeling of Anti-apoptotic Pathways in Prostate Cancer: Psychological Stress Triggers a Synergism Pattern Switch in Drug Combination Therapy
Source: PLoS Comput Biol. 2013 Dec 5;9(12):e1003358. doi: 10.1371/journal.pcbi.1003358 (PMC3854132; doi:10.1371/journal.pcbi.1003358)
Supplement: Table S3 — Quantification of experimental western blotting data. (PDF) [file pcbi.1003358.s005.pdf]

Para No.	Symbol	Value	Description
1	V_1	4.9412	maximal activation velocities of EGFR by EGF
2	K_1	1.0784	Michaelis activation coefficient of EGFR by EGF
3	V_2	3.7255	maximal activation velocities of Ras by EGFR
4	K_2	0.0588	Michaelis activation coefficient of Ras by EGFR
5	V_3	3.0588	maximal activation velocities of ERK by Ras
6	K_3	0.2549	Michaelis activation coefficient of ERK by Ras
7	V_4	2.2549	maximal activation velocities of KinaseX by ERK
8	K_4	1.4314	Michaelis activation coefficient of KinaseX by ERK
9	V_5	4.5294	maximal activation velocities of Rac by EGFR
10	K_5	0.2549	Michaelis activation coefficient of Rac by EGFR
11	V_6	4.9020	maximal activation velocities of PAK by Rac
12	K_6	1.0392	Michaelis activation coefficient of PAK by Rac
13	K_7	0.0392	Michaelis inhibition coefficient of PI3Kby LY
14	V_8	4.3333	maximal activation velocities of AKT by PI3K
15	K_8	1.6667	Michaelis activation coefficient of AKT by PI3K
16	V_9	2.9020	maximal activation velocities of cAMP by epinephrine
17	K_9	4.8824	Michaelis activation coefficient of cAMP by epinephrine
18	V_{10}	3.2745	maximal activation velocities of PKA by cAMP
19	K_{10}	4.1373	Michaelis activation coefficient of PKA by cAMP
20	V_{11}	3.8235	maximal activation velocities of CREB by PKA
21	K_{11}	0.0196	Michaelis activation coefficient of CREB by PKA
22	V_{12a}	4.4314	maximal activation velocities of S112BAD by PKA
23	K_{12a}	1.8431	Michaelis activation coefficient of S112BAD by PKA
24	V_{12b}	4.9216	maximal activation velocities of S112BAD by KinaseX
25	K_{12b}	2.0000	Michaelis activation coefficient of S112BAD by KinaseX
26	V_{12c}	0.8039	maximal activation velocities of S112BAD by PI3K
27	K_{12c}	0.1569	Michaelis activation coefficient of S112BAD by PI3K
28	V_{12d}	1.4118	maximal activation velocities of S112BAD by AKT
29	K_{12d}	4.5098	Michaelis activation coefficient of S112BAD by AKT
30	V_{13a}	2.7255	maximal activation velocities of S136BAD by PAK
31	K_{13a}	2.1765	Michaelis activation coefficient of S136BAD by PAK
32	V_{13b}	3.6863	maximal activation velocities of S136BAD by AKT
33	K_{13b}	3.0392	Michaelis activation coefficient of S136BAD by AKT
34	d_1	0.4510	Dephosphorylation rate of EGFR

35	d_2	1.1176	Dephosphorylation rate of Ras
36	d_3	0.7059	Dephosphorylation rate of ERK
37	d_4	0.4510	Dephosphorylation rate of KinaseX
38	d_5	3.6094	Dephosphorylation rate of Rac
39	d_6	2.4038	Dephosphorylation rate of PAK
40	d_7	1.0000	Dephosphorylation rate of PI3K
41	d_8	1.6250	Dephosphorylation rate of AKT
42	d_9	0.4933	Dephosphorylation rate of cAMP
43	d_{10}	0.6374	Dephosphorylation rate of PKA
44	d_{11}	3.7500	Dephosphorylation rate of CREB
45	d_{12}	1.9023	Dephosphorylation rate of S112BAD
46	d_{13}	0.9126	Dephosphorylation rate of S136BAD
